# Positionspapier der DGKL und der DIVI zu den Anforderungen an die Laboratoriumsmedizin in der Intensiv- und Notfallmedizin

**DOI:** 10.1007/s00063-024-01203-2

**Published:** 2024-11-05

**Authors:** Christian Waydhas, Carsten Hermes, Oliver Kumpf, Haitham Mutlak, Michael Spannagl, Felix Walcher, Peter B. Luppa, DIVI, DGKL

**Affiliations:** 1 Klinik für Unfall‑, Hand- und Wiederherstellungschirurgie, Universitätsklinikum Essen, Universität Duisburg-Essen, Hufelandstr. 55, 45147 Essen, Deutschland; 2 Hochschule für Angewandte Wissenschaften, Hamburg (HAW Hamburg), Alexanderstr. 1, 20099 Hamburg, Deutschland; 3 Studiengang „Erweiterte Klinische Pflege M.Sc und B.Sc.“, Akkon Hochschule für Humanwissenschaften, Berlin, Deutschland; 4 Klinik für Anästhesiologie m. S. operative Intensivmedizin, Campus Charité Mitte and Campus Virchow-Klinikum, Charité – Universitätsmedizin Berlin, Corporate Member of Freie Universität Berlin, Humboldt-Universität zu Berlin and Berlin Institute of Health, 10117 Berlin, Deutschland; 5 Klinik für Anästhesiologie, Intensivmedizin und Schmerztherapie, Sana Klinikum Offenbach, Offenbach, Deutschland; 6 Institut für Laboratoriumsmedizin, Ludwig-Maximilians-Universität, München, Deutschland; 7 Universitätsklinik für Unfallchirurgie, Universitätsmedizin Magdeburg, Magdeburg, Deutschland; 8 Institut für Klinische Chemie und Pathobiochemie, Klinikum rechts der Isar, Technische Universität München, München, Deutschland; 9 Deutsche interdisziplinäre Vereinigung für Notfall- und Intensivmedizin, Berlin, Deutschland; 10 Deutsche Gesellschaft für Klinische Chemie und Laboratoriumsmedizin, Bonn, Deutschland

**Keywords:** Intensivstation, Notaufnahme, Laborparameter, Point-of-Care-Testung (POCT), Zentrallabor, Intensive care unit, Emergency department, Laboratory parameter, Point-of-care testing (POCT), Central laboratory

## Abstract

**Hintergrund und Ziele:**

Die zeitgerechte Bestimmung und Bewertung von Laborparametern bei Patienten mit akuten lebens- oder organbedrohlichen Erkrankungen und Erkrankungszuständen in der Notaufnahme oder auf Intensivstationen kann für die Diagnosestellung, den Therapiebeginn und das Ergebnis essenziell sein. Ziel des Positionspapiers ist es, die zeitlichen Anforderungen an die Bereitstellung von labormedizinischen Ergebnissen in der Notfall- und Intensivmedizin zu definieren. Aus der Dringlichkeit lassen sich Anforderungen an Point-of-Care-Testung (POCT) und (zentrales) Labor ableiten.

**Methodik:**

Expertengruppen aus der Deutschen Gesellschaft für Klinische Chemie und Laboratoriumsmedizin (DGKL) und der Deutschen Interdisziplinäre Vereinigung für Intensiv- und Notfallmedizin (DIVI) entwickelten unter Nutzung von nationalen und internationalen Leitlinien, Reviewartikeln und Originalarbeiten eine Einteilung zur Dringlichkeit von Laborbestimmungen sowie Empfehlungen zu den erforderlichen Rahmenbedingungen und zur Qualitätssicherung.

**Ergebnisse:**

Es werden 3 Stufen der Dringlichkeit für die Bestimmung der gängigsten Laborparameter anhand der Turnaround Time definiert: Notfall 1, mit einer Turnaround Time von maximal 15 min; Notfall 2, mit einer Turnaround Time von maximal 60 min; dringlicher Fall, mit einer Turnaround Time innerhalb von 4 h. Zusätzlich wird eine Empfehlung zur Bereitstellung der Ergebnisse zur Hauptvisite auf der Intensivstation und der Notaufnahme gegeben.

**Schlussfolgerungen:**

Die Empfehlungen erlauben, die organisatorischen und apparativen Regelungen für jedes Krankenhaus anhand der medizinischen Anforderungen an die Dringlichkeit auszurichten.

## Abstract (en)

**Background and objectives:**

The timely determination and evaluation of laboratory parameters in patients with acute life- or organ-threatening diseases and disease states in the emergency room or intensive care units can be essential for diagnosis, initiation of therapy, and outcome. The aim of the position paper is to define the time requirements for the provision of laboratory results in emergency and intensive care medicine. Requirements for point-of-care testing (POCT) and the (central) laboratory can be derived from the urgency.

**Methods:**

Expert groups from the DGKL (Deutsche Gesellschaft für Klinische Chemie und Laboratoriumsmedizin) and DIVI (Deutsche Interdisziplinäre Vereinigung für Intensiv- und Notfallmedizin) developed a classification about the urgency for the determination of laboratory parameters as well as recommendations on the necessary organizational framework and quality assurance measures using national and international guidelines, review articles, and original papers.

**Results:**

Three levels of urgency are defined, based on the turnaround time of the most common laboratory parameters: emergency 1, with a turnaround time of no more than 15 min; emergency 2, with a turnaround time of a maximum of 60 min; urgent case, with a turnaround time within 4 h. In addition, a recommendation is made when to provide the results for the main ward rounds in the intensive care unit and the emergency department.

**Conclusions:**

The recommendations allow the organizational and technical regulations for each hospital to be aligned with the urgency of the provision of the test results to the medical team based on the medical requirements.

## 1 Einführung

Die Bestimmung und Bewertung von Laborparametern aus dem Blut bzw. aus anderen Körperflüssigkeiten oder Sekreten von Patienten mit akuten lebens- oder organbedrohlichen Erkrankungen und Erkrankungszuständen in der Notaufnahme von Krankenhäusern oder auf Intensivstationen ist für die Diagnosestellung und für die Verlaufskontrolle eine essenzielle, und oft entscheidende Voraussetzung.

## 2 Ziele des Positionspapiers

Ziel dieser Publikation ist es, die zeitlichen Anforderungen an die Bereitstellung von labormedizinischen Ergebnissen in der *Notfall- und Intensivmedizin* aus Anwendersicht zu definieren. Neben dem Bedarf an einer Optimierung der Zeitfenster zwischen Klinikaufnahme und Entscheidung zur Probengewinnung bzw. zwischen Befundkenntnisnahme und konsekutiver Diagnosestellung oder Handlung wird im Folgenden in erster Linie auf die Turnaround Time (TAT, Definition s. unten) fokussiert. Aus dem dafür jeweils resultierenden Zeitfenster ergeben sich Konsequenzen nicht nur für die Bestimmungsmethode, sondern ganz besonders für die organisatorischen Anforderungen. Hier steht die Zeitdauer für den Probentransport und den Beginn der Analyse im Mittelpunkt.

Eine zentrale Frage ist dabei der Bedarf, die Notwendigkeit und die Realisierbarkeit von Point-of-Care-Testung(POCT)-Analyseverfahren im Vergleich zu Verfahren, die ein zentrales klinisches Labor nutzen. Weiterhin sollen daraus Schlüsse gezogen werden, ob ein Labor im gleichen Gebäude bzw. Gelände oder extern (sei es an einem anderen Standort des Krankenhauses oder in einem externen Labor) geeignet sein kann.

*Die Empfehlungen betreffen konkret die klinisch- chemische Labordiagnostik. Mikrobiologische (Schnell-)Diagnostik, transfusionsbezogene Diagnostik und andere Verfahren, die überwiegend durch Präsenzlabore geleistet werden, sind nicht Gegenstand dieses Positionspapiers. Für solche Verfahren, wie die mikrobiologische (Schnell-)Diagnostik oder die transfusionsbezogene Diagnostik, gelten die Zeitvorgaben und Anforderungen aus den entsprechenden Leitlinien.*

## 3 Definitionen

**Abb. 1:**
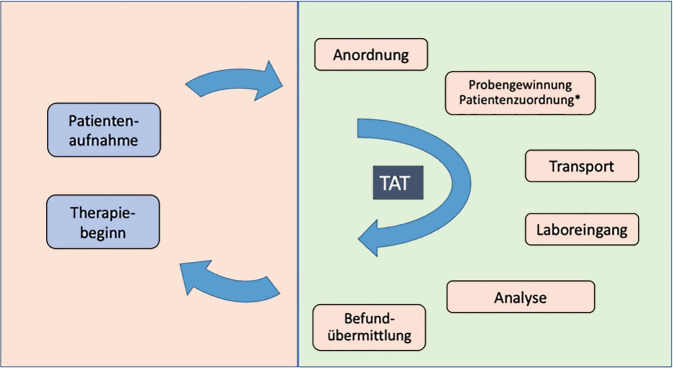
Zeitkomponenten der Labordiagnostik. Die Gesamtzeit beinhaltet den Zeitraum von der Patientenaufnahme bis zur Therapieeinleitung auf Basis eines Laborbefunds. Die Die TAT beschreibt den Zeitraum von der Anordnung der Probenabnahme bis zum vorliegenden Befund (*grün unterlegt*). Die *rot unterlegten* Zeitkomponenten sollten im Prozess der Notfallbehandlung so kurz wie möglich gehalten werden. Sie sind aber nicht Bestandteil der Betrachtungen dieses Positionspapiers. * Die eindeutige Patientenzuordnung ist Voraussetzung für die korrekte Laborwertzuordnung. *TAT* Turnaround Time

Für die TAT gibt es eine Vielzahl unterschiedlicher Definitionen [1]. Insbesondere bestehen oft unterschiedliche Auffassungen zwischen Labormedizinern und klinischen Medizinern [2–4]. Da es in dem vorliegenden Text um die klinische bzw. Patientensicht geht, wird die folgende Definition verwendet (Abb. 1):

Unter der TAT wird die Zeit zwischen der Anforderung des Tests und dem Erhalt des Ergebnisses verstanden [5, 6]. Dies umfasst die Zeitpunkte bzw. Zeitspannen für die folgenden Aktivitäten:

- Anordnung der Untersuchung,
- Probengewinnung mit eindeutiger Zuordnung zum Patienten,
- Transport,
- Probenannahme,
- Analyse,
- Befundübermittlung.

Darüber hinaus ist im Gesamtprozess auch die Zeit zwischen Eintreffen des Patienten und der Entscheidung zur Laborbestimmung einzubeziehen und zu optimieren. Dafür ist eine standardisierte Ersteinschätzung der eintreffenden Patienten durch Fachpflege- oder ärztliches Personal Voraussetzung. Sobald eine sofortige^1^ oder sehr dringliche^2^ Behandlungsnotwendigkeit festgestellt worden ist, soll die Indikation zur ggf. erforderlichen umgehenden Blutentnahme gestellt werden, deren Umfang von der Verdachtsdiagnose abhängt. Dafür sollten hausinterne Standards oder SOP entwickelt und umgesetzt werden, die für das betreuende Fachpersonal als Handlungskorridore dienen [7]. Eine solche SOP kann Aspekte wie die Gewinnung der Blutprobe, der Umfang der Bestimmungen sowie die Weiterleitung der Probe ins Labor oder die Durchführung der POCT-Messung beinhalten, ebenso wie ggf. erforderliche engmaschige Verlaufskontrollen.

Gleichermaßen ist nach der Übermittlung der Laborergebnisse die Zeit bis zur Bewertung durch Entscheidungspersonen und die Entscheidung zu einer Handlung zu berücksichtigen und optimal zu gestalten. Aufgrund einer permanenten Präsenz von Pflegefachpersonen und Ärzten in der Notaufnahme bzw. auf der Intensivstation bei Patienten mit hoher Behandlungsdringlichkeit bzw. -priorisierung sollte diese Zeit keine maßgebliche Verlängerung über die TAT hinaus verursachen.

POCT (auch patientennahe Sofortdiagnostik genannt): Es gibt eine Reihe unterschiedlicher Definitionen, was unter POCT verstanden werden kann. Konsensus besteht bei folgenden Merkmalen [5]:

1. Durchführung von Laboruntersuchungen in unmittelbarer Nähe zum Patienten,
2. Durchführung von Laboruntersuchungen außerhalb eines Zentral- oder Satellitenlaboratoriums,
3. keine Probenvorbereitung, d. h. meist Vollblut als Untersuchungsmaterial,
4. kein Pipettieren^3^,
5. „Ready-to-use“-Reagenzien, z. B. als Kassetten oder „unit-use devices“,
6. spezielle Messgeräte, die nur für die Einzelprobenmessung vorgesehen sind oder nur für die Einzelprobenmessung eingesetzt werden,
7. keine spezielle labormedizinische Qualifikation für die Messgerätebedienung notwendig,
8. rasche Verfügbarkeit der Ergebnisse,
9. unmittelbare Ableitung einer Diagnose bzw. von therapeutischen Konsequenzen aus den Ergebnissen.

Zentrallabor im Haus/Gelände: Das Zentrallabor befindet sich im gleichen Gebäude oder in einem Gebäude auf dem gleichen Gelände bzw. am gleichen Standort.

Zentrallabor an einem anderen Standort oder einem externen Labor: Hier ist das Labor außerhalb des Standorts lokalisiert und kann nur über öffentliche Verkehrswege erreicht werden.

Darüber hinaus sind weitere Organisationsformen für die Erbringung von Laborleistungen existent oder denkbar (z. B. dezentrale Analyseautomaten). Es ist im Rahmen dieses Positionspapier jedoch nicht vorgesehen, solche aufzuzählen oder zu bewerten. Für diese müsste im Einzelfall geprüft werden, ob die im Folgenden definierten Anforderungen sinngemäß erfüllt werden können.

## 4 Übergeordnete Anforderungen

Im Rahmen von Notfällen oder akuten schweren Störungen von Körper- und Organfunktionen ist naturgemäß eine hohe Dringlichkeit gegeben. Diese hohe Dringlichkeit besteht demgemäß auch und für die Bereitstellung von Diagnostik und von Ergebnisse dieser Diagnostik – so auch (u. a.) für die klinischen Laborparameter.

*In Krankenhäusern mit einer Notaufnahme oder einer Intensivstation ist die volle Betriebszeit für die Bestimmung der Notfalllaborparameter 24/7 (24 h am Tag, an jedem Tag) innerhalb der vorgegebenen TAT zu gewährleisten.*

Dies gilt unabhängig davon, ob POCT eingesetzt wird oder ein (zentrales) Labor vorhanden ist, und unabhängig davon, wo das (zentrale) Labor lokalisiert ist. Diese Anforderung besteht für alle Krankenhäuser mit Notaufnahmen (entsprechend G‑BA-Stufe, ab Basisnotfallversorgungversorgung [8]) oder Intensivstationen [9].

Die Dringlichkeit ergibt sich aus der Schwere der Erkrankung oder des Krankheitszustands und dessen zeitlicher Dynamik, die umgehende Behandlungsmaßnahmen erfordern.

Aus der Dringlichkeit ergibt sich die Zeitspanne (TAT laut obiger Definition), innerhalb derer das Ergebnis von Laboruntersuchungen im Notfall vorliegen soll.

Die POCT hat den klaren Vorteil, dass die TAT stark verkürzt werden kann. Nur die patientennahe Sofortdiagnostik ermöglicht insbesondere für die Erkennung und Behandlung (potenziell) lebensbedrohlicher Zustände eine Befundbereitstellung innerhalb eines so engen Zeitfensters, wie es der klinischen Situation angemessen ist. Für viele andere Parameter kann aber auch ein Zentrallabor im Haus oder auf dem Gelände eine ausreichend schnelle Bereitstellung der Ergebnisse gewährleisten, sofern die dafür erforderlichen organisatorischen Abläufe sichergestellt sind. Prinzipiell kann auch ein Zentrallabor an einem anderen Standort oder ein externes Labor für bestimmte Parameter, die in die Gruppe der dringlichen Fälle fallen, ausreichend sein, solange die vorgegebenen Zeitspannen sicher und zu jeder Zeit eingehalten werden können.

Obwohl die Anforderung an eine eindeutige Zuordnung einer Probe zu einem Patienten für alle Laborproben gilt, ist diese auch bei den POCT-Verfahren sorgfältig sicherzustellen, um Proben- oder Patientenverwechslungen, gerade in der Notfallsituation, zu vermeiden.

Für die Bewertung und Anwendung von POCT-Verfahren darf der präanalytische Aufwand in der Probenverarbeitung nicht vernachlässigt werden. Neben der Blutabnahme und dem Befüllen des Analysegeräts können auch Tätigkeiten wie Mischen der Probe, Befüllung spezieller Probenröhrchen oder Pipettierschritte erforderlich sein. Bei einer komplexen Probengewinnung (z. B. Liquorpunktion) ist der Aufwand sowohl, um die Zeit zwischen Indikation und Durchführung gering zu halten, als auch bedingt durch die Schwierigkeit der Maßnahme selbst besonders hoch.

Eine erhöhte Anforderung ergibt sich im Weiteren durch das Ablesen eines Ergebnisses und ggf. die händische Eingabe in die EDV. Ein erhöhter Aufwand besteht beispielsweise auch dann, wenn für die POCT-Diagnostik mehr als ein Gerät zu bedienen ist.

**Abb. 2:**
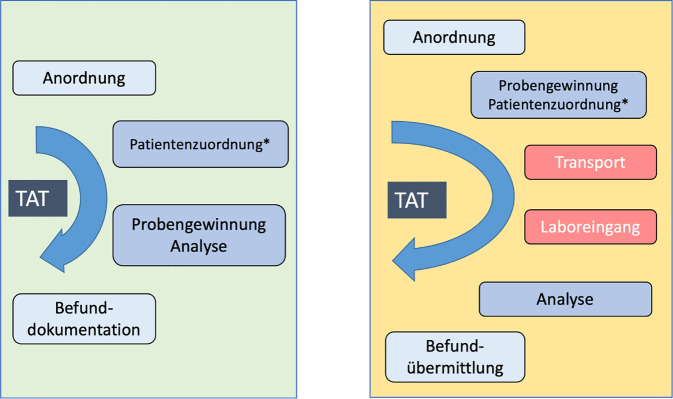
Zeitkomponenten der Labordiagnostik mit und ohne POCT. Die POCT-Diagnostik zeichnet sich durch eine deutlich einfachere Transportlogistik (zusätzliche Schritte in der Abbildung als *rote* Kästchen kenntlich) aus und damit eine verkürzte TAT. Die zahlenmäßig weniger vorhandenen Arbeitsschritte und Übergabestellen machen die POCT-Diagnostik weniger anfällig für qualitätsrelevante Fehler. * Die eindeutige Patientenzuordnung ist Voraussetzung für die korrekte Laborwertzuordnung. *TAT* Turnaround Time

In der Systematik des Probenlaufs in der medizinischen Labordiagnostik sind die prä- bzw. postanalytischen Phasen (extraanalytischen Phasen) Teil der TAT. In der wissenschaftlichen Aufarbeitung dieser Themen wurde in vielen Fehleranalysen klar herausgearbeitet, dass die weit überwiegende Zahl der Fehler bis zur Identifikation der Probe im Labor bzw. dann bei der Ergebnismitteilung und Interpretation stattfinden. Nur etwa 10 % der Fehler betreffen die analytische Phase selbst [10]. Hier ergeben sich in der POCT-Situation auf Grund des fortwährenden Kontakts zwischen Patienten und Behandlungsteam deutlich einfachere Abläufe. Trotzdem benötigen Probengewinnung und Interpretation der POCT-Resultate höchste Aufmerksamkeit der Durchführenden (Abb. 2).

Bei POCT entfällt in der Regel eine aufwändige Transportlogistik, die bei Zentrallaboren im Gebäude/Gelände und noch viel mehr bei Laboren an einem anderen Standort oder einem externen Labor aufwändig sein und zu starken Verzögerungen führen kann. Dies kann bei instabilen Analyten (z. B. pO_2_ oder pCO _2_) essenziell sein. Weiterhin besteht bei POCT ein geringerer Personalbedarf, da keine Personalvorhaltung ausschließlich für die Labortätigkeiten erforderlich ist, sofern andernorts (Labor‑)Personal eingespart werden kann. Die Bindung von Personal vor Ort darf aber nicht vernachlässigt werden (siehe weiter unten).

Allerdings sind bei POCT die Materialkosten pro Bestimmung in der Regel höher. Es soll hier aber keine ökonomische Betrachtung erfolgen, da eine Abwägung der Personalkosten gegenüber den Materialkosten an verschiedenen Krankenhäusern mit unterschiedlichen Organisationsstrukturen differenziert betrachtet werden muss. So können die Vorhaltungen für den Probentransport unterschiedlich sein (z. B. Transportdienst vs. Rohrpost) oder das eingesetzte Personal wird neben der Labortätigkeit beispielsweise auch für Aufgaben in der mikrobiologischen Diagnostik oder der Transfusionsmedizin eingesetzt. Bezüglich weiterer indirekter Kosten z. B. in der Qualitätskontrolle siehe weiter unten.

*Entscheidend, sowohl für die organisatorischen Lösungen und Regelungen als auch für die ökonomischen Aspekte, muss sein, dass die klinisch-medizinischen Anforderungen, wie sie sie sich aus der Dringlichkeit ergeben, erfüllt werden.*

Aus medizinischer Sicht sollte bei der Bewertung der Laborergebnisse beachtet werden, dass es systematische Unterschiede zwischen den Ergebnissen einer POCT-Analyse und einer Untersuchung aus dem Zentrallabor geben kann. Diese sollten den Anwendern bekannt sein, um Fehlinterpretationen zu vermeiden.

Neben dem Zeitfaktor müssen in jedem Falle die Qualität der Analyse und die Datensicherheit sichergestellt sein (siehe weiter unten).

Für die erfolgreiche Implementierung von POCT-Methoden im Bereich der Intensiv- und Notfallmedizin (einer Einrichtung) sollte gemeinsam mit dem Zentrallabor ein integratives Konzept entwickelt werden. Dabei sollten die Parameter, die die POCT umfassen soll, definiert werden und geeignete Standorte für die entsprechenden Geräte festgelegt werden.

Für Leserinnen und Leser, die weitergehende und umfassendere Hinweise für die Implementierung von POCT benötigen, verweisen wir an ein von Luppa und Junker herausgegebenes Fachbuch über POCT [5].

## 5 Dringlichkeit von Laboruntersuchungen

Die Dringlichkeit, mit der ein bestimmtes Laborergebnis vorliegen soll, ergibt sich aus der Akuität und Bedrohlichkeit des Erkrankungszustands sowie dem Erfordernis und der Möglichkeit eines zeitnahen Handelns. Diese Dringlichkeit lässt sich in vielen Fällen nicht wissenschaftlich sicher belegbar in exakten Minuten- oder Stundenwerten angeben. Allerdings gibt es eine abstrakte abgestufte Dringlichkeit, die sich aus der klinischen Erfahrung und aus Expertenmeinungen ableiten lässt. Indirekt lässt sich eine Dringlichkeit auch aus den Ersteinschätzungsinstrumenten ableiten [11]. Typischerweise sind Patienten der beiden dringlichsten Behandlungsprioritäten sofort bzw. innerhalb von wenigen bis maximal 10–15 min zu behandeln, jene der dritten Behandlungspriorität innerhalb von 30–60 min. Analog können diese Zeitspannen auch für das Vorliegen der Laborergebnisse bei solchen Patienten angenommen werden. Sofern keine wissenschaftlich gesicherten Daten zur Verfügung stehen, wird auf Expertenmeinungen im Rahmen von nationalen und internationalen Leitlinien und Empfehlungen zurückgegriffen. Begründungen für deren Empfehlung werden dort ausgeführt und brauchen im Text nicht mehr wiederholt oder diskutiert werden. *Somit entsprechen die zeitlichen Vorgaben einer sehr starken Empfehlung. In ungünstigen Einzelfällen unter realen Bedingungen ist allerdings eine 100* *%-Einhaltung dieser Vorgaben unrealistisch und auch bei einer Behandlung und Behandlungsabläufen im Rahmen der State of the Art kann es unvermeidbare Abweichungen nach oben geben, ohne dass dies eine fehlerhafte oder ungenügende Behandlung bedeutet. Diese sollten dennoch erklärend dokumentiert und im Sinne einer hausinternen Qualitätsverbesserung besprochen werden, damit die Abweichungen so gering wie möglich ausfallen.*

Die einzelnen Messverfahren unterliegen einer methodeninhärenten Analysedauer, die unabhängig vom Wunsch, ein Ergebnis schneller vorliegen zu haben, nicht oder nur mit einem nicht vertretbaren ökonomischen Aufwand verkürzt werden kann. Zukünftige verfahrenstechnische oder analytische Verbesserungen und Neuerungen können eine Neubewertung der Dringlichkeit einzelner Parameter erforderlich machen.

### 5.1 Notfall 1: Turnaround Time von maximal 15 min

Hier handelt es sich um Parameter, die Störungen beschreiben, die bei pathologischen Abweichungen ein sofortiges Handeln erforderlich machen, da ansonsten schwerste organ- und lebensgefährdenden Komplikationen drohen.

Für die Parameter dieser höchsten Dringlichkeitsstufe soll für Notfallsituationen eine TAT von maximal 15 min eingehalten werden [12]. Allerdings kann bei perakuten Problemen, beispielsweise respiratorischen Zwischenfällen auf der Intensivstation, in der Notaufnahme oder im Operationssaal, dieses Zeitfenster zu lang und ein noch schnelleres Vorliegen des Messergebnisses angezeigt sein. Hier kann es auch um noch kürzere Zeitspannen gehen.

Es soll sichergestellt sein, dass beispielsweise auch bei einer gerade laufenden Kalibrierung eines Blutgasanalyse(BGA)-Geräts und/oder gleichzeitig mehr als einer dringenden Analyse dieses enge Zeitfenster eingehalten wird. Es ist also zu erwägen, POCT-Geräte im Backup-Modus zu betreiben. Um darüberhinausgehende Verzögerungen, insbesondere Wegezeiten u. ä., auf einem Minimum zu halten, ist es in der Regel erforderlich, die räumliche Nähe der Analysegeräte zum Behandlungsort sicherzustellen.

Daraus ergeben sich 2 Anforderungen, um eine Einhaltung der Zeitvorgaben sicherzustellen:

1. Dieses Zeitfenster lässt sich in aller Regel nur mittels BGA-Geräte sicherstellen. Dabei handelt es sich nicht um BGA-Geräte im engeren Sinne, sondern um solche, die auch die gleichzeitige Bestimmung aller der in Tab. 1 genannten Parameter erlauben.
2. Ein geeignetes BGA-Gerät wird auf der Intensivstation *und* in der zentralen Notaufnahme ständig einsatzbereit vorgehalten. Dies dient einerseits der Reduzierung der Wegstrecken, andererseits ist die Redundanz bei technischen Defekten eines Geräts notwendig. Die Überlegungen dieses Beitrags waren primär nicht auf den Operationsbereich ausgerichtet. Aber auch dort ist bei akuten Zwischenfällen eine entsprechend schnelle POCT-Diagnostik notwendig. In aller Regel soll auch im OP-Bereich ein BGA-Gerät zusätzlich aufgestellt und ohne Ein- und Ausschleusung des Personals erreichbar sein. In kleinen Häusern, z. B. mit Basisnotfallversorgung, oder Fachkliniken mit selektioniertem Patientenklientel und einer sehr niedrigen Rate an intraoperativen Zwischenfällen kann das BGA-Gerät auch in einer räumlich eng benachbarten Intensivstation stehen, sofern unter Berücksichtigung von Einschleusungs- und Ausschleusungszeiten und der Wegstrecke die TAT von 15 min eingehalten werden kann.

**Tab. 1:** Laborparameter, bei denen eine Turnaround Time (TAT) zwischen Anordnung der Untersuchung und Vorliegen der Ergebnisse innerhalb von 15 min erreicht werden soll^a^

Parameter	Personalbindung^b^	Kommentar	Literatur
Blutgasanalyse incl. CO-Hb + Säure-Basen-Haushalt	Gering	–	[8, 12–14]
Laktat	Gering	–	[13–15]
Glukose	Gering	Kapillarblutabnahme nicht indiziert	[14]
Kalium	Gering	–	[8, 12, 14]
Kalzium ionisiert	Gering	–	[13]
Hämoglobin	Gering	–	[14]

^a^Werden diese Untersuchungen als geplante Bestimmungen im Rahmen der Routine bzw. außerhalb von akuten Notfallsituationen angeordnet, gelten die Zeitvorgaben aus den Tab. 2 oder 3

^b^Geringer Aufwand vor Ort: Blutabnahme, Befüllen des Geräts und Eingabe der Patienten-ID

Auch im Notfall und für alle POCT-Geräte ist sicherzustellen, dass die Ergebnisse jeder POCT-Analyse zuverlässig und sicher dokumentiert werden. Dies lässt sich mit hinreichender Sicherheit nur mittels fester Anbindung der BGA-Geräte an ein Labor-EDV-System, ein PDMS, ein Krankenhausinformationssystem oder eine vergleichbare IT-Lösung erreichen. Ein Ausdruck auf einem nichtdokumentensicheren Papier (z. B. Thermopapier) ist dazu nicht ausreichend. Auch für einfache Messgeräte (z. B. ACCU-Chek® und vergleichbare), sollten sie in diesen High-care-Bereichen überhaupt eingesetzt werden, muss gewährleistet sein, dass eine entsprechende Dokumentation erfolgt. Dies gilt gleichermaßen für Untersuchungen mit diversen Teststreifen (Stix) oder Antigenschnelltests u. ä. Verfahren, für die eine Implementierung in bestehende Dokumentationssysteme bestehen soll.

Die Anforderungen an das Personal, das das BGA-Gerät bedient, sind insgesamt als niedrig einzuschätzen und können durch eine Geräteeinweisung gemäß der jeweils gültigen Medizinproduktbetreiberverordnung (MPBetreibV) sichergestellt werden. Der Zeitaufwand für die Messung ist ebenfalls gering. Das Ergebnis muss ohne Verzögerung dem (be)handelnden Fachpersonal zur Verfügung stehen.

### 5.2 Notfall 2: Turnaround Time von maximal 60 min

Hier handelt es sich um Parameter, die Störungen beschreiben, die bei starken pathologischen Abweichungen ein umgehendes Handeln erforderlich machen, da ansonsten schwerste organ- und lebensgefährdenden Komplikationen drohen, bei denen es aber in der Regel nicht auf wenige Minuten ankommt.

Es besteht ein breiter Konsens, dass die TAT für diese Parametern bei Notfällen 60 min nicht überschreiten soll. Unabhängig von dieser Maximalzeit ist eine (ggf. deutlich) schnellere Bereitstellung von Ergebnissen in vielen Situationen wünschenswert, z. B. von Troponinen (T oder I) oder Gerinnungswerten (durch viskoelastische Tests). Das würde einen schnelleren und gezielteren Therapiebeginn (z. B. Verabreichung von Gerinnungsprodukten) ermöglichen oder unnötige Diagnostik, Therapie, Überwachung oder Inanspruchnahme von Ressourcen vermeiden. Bestimmte Parameter (z. B. Natrium, ionisiertes Kalzium) sollten in das Panel der BGA-Geräte integriert werden.

Für praktisch alle in Tab. 2 genannten Parameter (Ausnahme: Fibrinogen, quantitativ) stehen auch POCT-Verfahren zur Verfügung. Diese sind jedoch teilweise sehr teuer (z. B. Interleukin‑6, Prokalzitonin u. a.) oder bedürfen eines vergleichsweise hohen Personalaufwands vor Ort (z. B. bestimmte viskoelastische Tests). Dieser Personalaufwand kann in einem erhöhten Schulungsaufwand und/oder Zeitbedarf bei der Durchführung der Untersuchung selbst bestehen. Beide Aspekte werden insbesondere dann relevant, wenn mehrere unterschiedliche Geräte zu bedienen sind.

In der Regel kann die TAT von 60 min bei Nutzung eines Zentrallabors eingehalten werden. Ist dies (unabhängig von den Gründen) jedoch nicht möglich, sollen POCT-Verfahren eingesetzt werden, um die zeitgerechte Labordiagnostik sicherzustellen.

Für die Dokumentationssicherheit bei Einsatz von POCT gelten die oben gemachten Anforderungen.

**Tab. 2:** Laborparameter, bei denen eine Turnaround Time (TAT) zwischen Anordnung der Untersuchung und Vorliegen der Ergebnisse innerhalb von maximal 60 min erreicht werden soll^a^

Parameter	Personalbindung^b^	Kommentar	Literatur
Natrium	Gering	Mittels BGA-Gerät bestimmen	[8, 12, 14, 16]
Chlorid	Gering	–	–
Kalzium, ionisiert	Gering	Mittels BGA-Gerät bestimmen	[13]
Kreatinin	Gering	–	[8, 12, 17]
Magnesium	Gering	–	–
Kleines Blutbild (Leukozyten, Thrombozyten)	Gering	Auf mögliche Fehlermeldungen (Flags) achten	[8, 12, 13, 16]
Troponin T oder I	Gering	Für hochsensitive Tests Zentrallabor bevorzugen [18]	[8, 12, 14, 17–19]
aPTT, TZ, INR	Gering	–	[8, 12–14, 16, 20]
Fibrinogen	Gering	Keine POCT, kann durch viskoelastische Fibrinogenmessung ersetzt werden	[13, 20]
Viskoelastische Tests	Geräteabhängig Moderat bis hoch	–	[13]
D‑Dimer	Gering	–	[8, 12]
BNP	Gering	–	[8, 12, 17]
C‑reaktives Protein	Gering	Zumindest einer der beiden Tests (CRP, PCT)	[8, 12, 17]
Prokalzitonin	Gering	Zumindest einer der beiden Tests (CRP, PCT)	[14]
Interleukin‑6	Gering	Neonatologie, Pädiatrie	–
Antigene (COVID, Influenza)	Moderat	–	–
Drogenscreening (Urin)	Moderat	–	–
Urin-Teststreifen	Moderat	–	[14, 17]
Schwangerschaftstest (Urin)	Moderat	–	[14]

^a^Werden diese Untersuchungen als geplante Bestimmungen im Rahmen der Routine bzw. außerhalb von akuten Notfallsituationen angeordnet, gelten die Zeitvorgaben aus Tab. 3

^b^Geringer Aufwand: Blutabnahme, Befüllen des Geräts und Eingabe der Patienten-ID; moderater Aufwand: spezielle Probenvorbereitung, Ablesen des Ergebnisses und händische Eingabe in die EDV; moderater bis hoher Aufwand: zusätzliche zum BGA-Gerät zu bedienende Geräte oder Durchführung zusätzlicher analytischer Schritte (z. B. Pipettieren oder Probenaufbereitung)

### 5.3 Dringlicher Fall: Turnaround Time innerhalb von 4 h

Hier handelt es sich um Parameter, die Störungen beschreiben, die bei starken pathologischen Abweichungen ein schnelles Handeln erforderlich machen, da ansonsten schwere organ- und lebensgefährdende Komplikationen drohen oder eine notwendige Therapie verzögert wird, woraus eine verzögerte Wirksamkeit resultieren kann. In der Regel kommt es nicht auf Minuten an.

Es besteht ein breiter Konsens, dass die TAT für diese Parametern bei Notfällen 4 h nicht überschreiten darf [14]. Unabhängig von dieser Maximalzeit ist eine (ggf. deutlich) schnellere Bereitstellung von Ergebnissen in vielen Situationen wünschenswert. Dann könnten die Diagnosen früher gestellt werden und ein schnellerer und gezielterer Therapiebeginn möglich sein oder die organisatorischen Entscheidungen (Art der Überwachung, Ort der Weiterversorgung, stationäre oder ambulante Behandlung etc.) schneller getroffen und damit Ressourcen eingespart werden.

Mit aktuellen Analyseautomaten können die allermeisten der in Tab. 3 genannten Parameter innerhalb der erforderlichen TAT bestimmt werden. Die Anforderungen an das Personal vor Ort sind in der Regel gering. Ist die Einhaltung der TAT (unabhängig von den Gründen) jedoch nicht möglich, sollen POCT-Verfahren eingesetzt werden, um die zeitgerechte Labordiagnostik sicherzustellen. Für die Dokumentationssicherheit beim Einsatz von POCT gelten die oben gemachten Anforderungen.

**Tab. 3:** Laborparameter, bei denen eine Turnaround Time (TAT) zwischen Anordnung der Untersuchung und Vorliegen der Ergebnisse innerhalb von maximal 4 h erreicht werden soll oder die zu einem bestimmten Zeitpunkt zur Frühvisite vorliegen sollen

Parameter^a^	Personalbindung^b^	Kommentar	Literatur
*Elektrolyte*			
Kalzium, Gesamtkalzium	Gering	–	[17]
Phosphat	Gering	–	[17]
Differenzialblutbild	Gering	–	[17]
*Gerinnung*			
ACT	Moderat	POCT	–
Thrombozytenfunktion	Gering	–	–
Anti-Xa	Gering	–	–
Thrombininhibitoren	Gering	–	–
*Serumchemie*			
CK	Gering	–	[17]
Myoglobin	Gering	Rhabdomyolyse	–
Lipase oder Amylase	Gering	–	[14, 20]
LDH	Gering	–	[17, 20]
GOT	Gering	–	[14, 17, 20]
GPT	Gering	–	[14, 17, 20]
γ‑GT	Gering	–	[14, 17]
Alkalische Phosphatase	Gering	–	–
Bilirubin	Gering	–	[14, 17]
Harnstoff	Gering	–	[20]
Harnsäure	Gering	–	–
Albumin	Gering	–	–
Gesamteiweiß	Gering	–	–
Ammoniak	Moderat	–	–
*Hormone*			
Schilddrüsenhormone (TSH, FT_3_, FT_4_)	Gering	Im Notfall KM-Gabe auch ohne Kenntnis des TSH Wertes	[14, 17]
Cortisol	–	–	–
*Infektionsdiagnostik*			
HIV-Test 4. Generation	Gering	Laut BG-Richtlinien innerhalb von 2 h bzw. 4. Generation	[14, 21]
MRSA-Schnelltest	Moderat	–	–
Legionellenantigen (Urin)	Gering	–	–
Hämoccult	Moderat	–	–
*Diverse*			
Liquorzellzahl	Hoch	–	[14]
Alkohol	Gering	–	[14]
Ketonkörper im Kapillarblut	Gering	–	–
Paracetamol	Gering	–	[14, 20]
Intoxikationen	–	Nach lokalem Bedarf	–

^a^Die genannten Parameter beanspruchen keine Vollständigkeit. Bei spezialisierten Krankenhäusern oder Abteilungen können zusätzliche Laborbestimmungen erforderlich oder andere Werte nicht erforderlich sein. Die genannten Parameter sind in der Regel in allen Krankenhäusern, die mindestes in die Basisnotfallversorgung eingestuft sind, erforderlich. Krankenhäuser der erweiterten und umfassenden Notfallversorgung sollen neben den erforderlichen BGA-Geräten über ein Zentrallabor mit 24 h-Bereitschaft verfügen und in der Regel die Bestimmungen mit einer TAT deutlich unter der Maximalzeit von 4 h anbieten

^b^Geringer Aufwand: Blutabnahme, Befüllen des Geräts und Eingabe der Patienten-ID; moderater Aufwand: spezielle Probenvorbereitung, Ablesen des Ergebnisses und händische Eingabe in die EDV; moderater bis hoher Aufwand: zusätzliche zum BGA-Gerät zu bedienende Geräte oder Durchführung zusätzlicher analytischer Schritte (z. B. Pipettieren oder Probenaufbereitung); hoher Aufwand: aufwändige Probengewinnung

### 5.4 Bereitstellung der Ergebnisse zur Hauptvisite auf der Intensivstation und den Notaufnahmen

In Deutschland beginnt die Hauptvisite auf 91 % der Intensivstationen um 7.00 Uhr oder zwischen 7.00 und 8.00 Uhr [22]. Für die Planungen und Entscheidungen, die bei der Visite getroffen werden, sind die Laborwerte eine entscheidende Informationsquelle. Somit ist es notwendig, dass diese zum Visitenbeginn bereits vorliegen. Die TAT muss so bemessen sein, dass die Blutabnahme möglichst spät erfolgen kann, um die Nachtruhe der Intensivpatienten einerseits wenig zu beeinträchtigen und dass andererseits die Ergebnisse rechtzeitig vorliegen. Die Organisation und Leistungsfähigkeit des Labors sind entsprechend anzupassen.

## 6 Anforderungen an das Behandlungsteam

Aufgabe des Behandlungsteams ist es, die präanalytische Phase zu optimieren und Fehler zu minimieren. Bestimmungen, die durch präanalytische Fehler verfälscht sind, können neben einer relevanten Patientengefährdung erhebliche rechtliche und materielle Konsequenzen haben [23]. Fehlerhafte Präanalytik kann Wiederholungsmessungen notwendig machen, ggf. mit erneuter Punktion. Möglicherweise ist die Probe nicht ohne weiteres nochmals zu gewinnen. Dies ist z. B. bei einer Liquorentnahme patientenbelastend, aufwändig und u. U. mit Komplikationen vergesellschaftet. Noch gravierender ist es, wenn die Fehlbestimmung nicht oder zu spät bemerkt wird und bereits therapeutische oder invasive diagnostische Maßnahmen eingeleitet wurden.

Typische Probleme sind nach [5] beispielsweise:

- falsche oder fehlende Identifikation des Patienten, u. U. doppelte Patientenverwechslungen;
- fehlerhafte Probengewinnung (z. B. zu lange Stauung bei venöser Abnahme oder falsches Probenröhrchen);
- zu langsame Weiterverarbeitung der Probe (z. B. Liegenlassen von arteriellen Blutgasproben, Quetschen bei Kapillarblutentnahme);
- Hygienefehler bei Abnahme aus Verweilkathetern;
- Unkenntnis über die laufende Medikation (z. B. bei aPTT-Kontrolle oder ACT-Kontrolle Probenentnahme vom Arm mit einer Heparininfusion oder über einen heparinbestückten Katheter);
- eine mangelnde Einweisung in die Geräte kann zu Fehlbestimmungen oder Probenverlusten führen.

Eine (ggf. wiederholte) Schulung des Behandlungspersonals im Umgang mit Laborproben und den genutzten Messgeräten ist somit unerlässlich.

Bei der Bewertung des Aufwands für das Behandlungspersonal bei der Anwendung von POCT ist dieser zusätzliche Aufwand zu bedenken. Während die Blutabnahme, das Befüllen des Geräts und die Eingabe der Patienten-ID für alle Laboruntersuchungen, unabhängig ob per POCT oder ein Zentrallabor, gleich ist, bleibt der Aufwand für ein zusätzliches Ablesen des Ergebnisses und die händische Eingabe in die EDV im Rahmen einer POCT-Untersuchung (z. B. von Stix) moderat und ist Teil der Routineabläufe.

Der Aufwand steigt jedoch deutlich an, wenn mehrere POCT-Geräte zu bedienen sind (Befüllen der Probe in mehrere Geräte, umfangreichere Einweisungen, umfangreichere Fehlerbehebung usw.). Ein Pipettieren oder Zentrifugierung von Proben erfordert labortechnische Kenntnisse und Fertigkeiten, die geschult und geübt werden müssen. Solche Tätigkeiten sind außerdem fehleranfällig und zeitintensiv. Die dafür notwendigen Zeitspannen sind bei der Personalbemessung in Notaufnahmen und Intensivstationen bislang nicht berücksichtigt. Eine Einsparung von Laborpersonal zu Lasten des Personals am Patienten ist ohne personelle Verstärkung der Mitarbeiterinnen und Mitarbeiter in den besagten Bereichen nicht praktikabel.

## 7 Qualitätskontrolle, gesetzliche Rahmenbedingungen und Anforderungen

*Die Qualitätssicherung laboratoriumsmedizinischer Untersuchungen inklusive der POCT-Verfahren ist gemäß der „Richtlinie der Bundesärztekammer zur Qualitätssicherung laboratoriumsmedizinischer Untersuchungen“ (Rili-BÄK) für alle Einrichtungen verbindlich, in denen Laboranalysen im Rahmen der Heilkunde durchgeführt werden *[24]*. Die darin beschriebene Qualitätssicherung der POCT folgt dem Grundsatz, dass Laborbefunde am POC und im Zentrallabor im Sinne der Patientensicherheit eine vergleichbare analytische Zuverlässigkeit haben sollten. Dies bedeutet, dass die bewährten Qualitätsstandards der konventionellen Labordiagnostik auch für die POCT gelten. *Daher gibt es in der aktuellen Rili-BÄK von 2023* – mit einer Ausnahme – für POCT keine Sonderregelungen.*

Die Richtlinie ist in 2 Teile gegliedert:

Teil A: „Grundlegende Anforderungen an die Qualitätssicherung labormedizinischer Untersuchungen“. Dieser Teil gilt für alle Bereiche der labormedizinischen Diagnostik, d. h. für die klassischen Zentrallaboratorien im Krankenhaus, aber auch für Arztpraxen und die POCT-Diagnostik, und beschreibt die Implementierung eines umfassenden Qualitätsmanagementsystems (QMS). Für die POCT-Diagnostik sind folgende Punkte zu regeln:

1. Festlegung von Verantwortlichkeiten für das gesamte QMS am POC,
2. Organisation der POCT,
3. Vorschriften für die Präanalytik,
4. Vorschriften für die Durchführung der dezentralen Untersuchungen,
5. Vorschriften für die Postanalytik,
6. Schulung des Personals sowie Aufgaben und Verantwortlichkeiten bei der Durchführung der Qualitätskontrollen.

Der Teil B: „Spezielle Teile für laboratoriumsmedizinische Untersuchungen“ beschreibt das System der durchzuführenden Qualitätskontrollen. Alle quantitativen und qualitativen labormedizinischen Untersuchungen unterliegen der internen und – so weit in den Tabellen B1 und B2 der Rili-BÄK aufgeführt – der externen Qualitätssicherung. Bei der Durchführung der Qualitätskontrolle gibt es mit einer Ausnahme (bei Verwendung einer Kartusche oder eines Unit-use-Teststreifens) keine Sonderregelungen für POCT-Verfahren. Dies gilt auch für die Beurteilung der Kontrollprobeneinzelmessung. Die zulässige Abweichung des Messwerts bei der internen und externen Qualitätskontrolle ist für Serum/Plasma und Blut identisch, unabhängig davon, ob die Werte am POC oder im Zentrallabor erhoben wurden.

Um die Vorgaben der Rili-BÄK zu erfüllen, empfiehlt sich in einem Krankenhaus oder einem Krankenhausverbund die Einrichtung einer *POCT-Koordinationsstelle*, die die verschiedenen Organisationseinheiten eines Krankenhauses (Notaufnahme, Intensivstationen, OP-Bereiche etc.) zusammen mit dem Zentrallabor für die POCT-Analytik zu einer einzigen Organisationseinheit zusammenfasst. Dies muss durch eine schriftliche Dienstanweisung seitens der Krankenhausleitung geschehen. Die Koordinationsstelle entwirft für die Implementierung von POCT in einem Krankenhaus unter Beteiligung des Zentrallabors ein integratives Konzept. Ein solches Konzept koordiniert alle wesentlichen Interessengruppen und Belange dieser Diagnostik und gewährleistet die Qualität des labormedizinischen Befunds durch die Umsetzung der von der Rili-BÄK vorgeschriebenen Maßnahmen zum QMS.

Zu beachten ist, dass die Organisation und Durchführung der Qualitätskontrollen auch dann nach Rili-BÄK stattfinden, wenn das krankenhauseigene Labor geschlossen oder ausgelagert wurde. Dies schließt die Wartung und gegebenenfalls Reparatur der Messgeräte ein, falls eine medizintechnische Abteilung nicht existiert oder nicht in der Lage ist, die Wartung bzw. Reparatur durch das entsprechend geschulte Pflegefachpersonal zu übernehmen. Darüber hinaus beinhaltet dies die Überwachung der auf der Station vorhandenen Vorräte an Reagenzien und notwendigen Hilfsmitteln.

Personen, die die POCT-Geräte bedienen, sowie die Verantwortlichen für die Probenentnahme müssen regelmäßig in der Handhabung und den Besonderheiten der jeweiligen Geräte geschult werden. Auch dafür trägt die POCT-Koordination die Verantwortung.

## Footnotes

1 Lebensbedrohliche Situation

2 Notfall, der lebensbedrohlich werden kann

3 Bei bestimmten POCT-Verfahren können einzelne Merkmale abweichen (z. B. Pipettieren bei Gerinnungsanalysen mit Thrombozytenfunktionstestungen oder bei Antigenschnelltests).
